# Supplementary figures and images for: Shaping and interpretation of Dpp morphogen gradient by endocytic trafficking
Source: PLoS Genet. 2025 Jul 14;21(7):e1011766. doi: 10.1371/journal.pgen.1011766 (PMC12310002; doi:10.1371/journal.pgen.1011766)

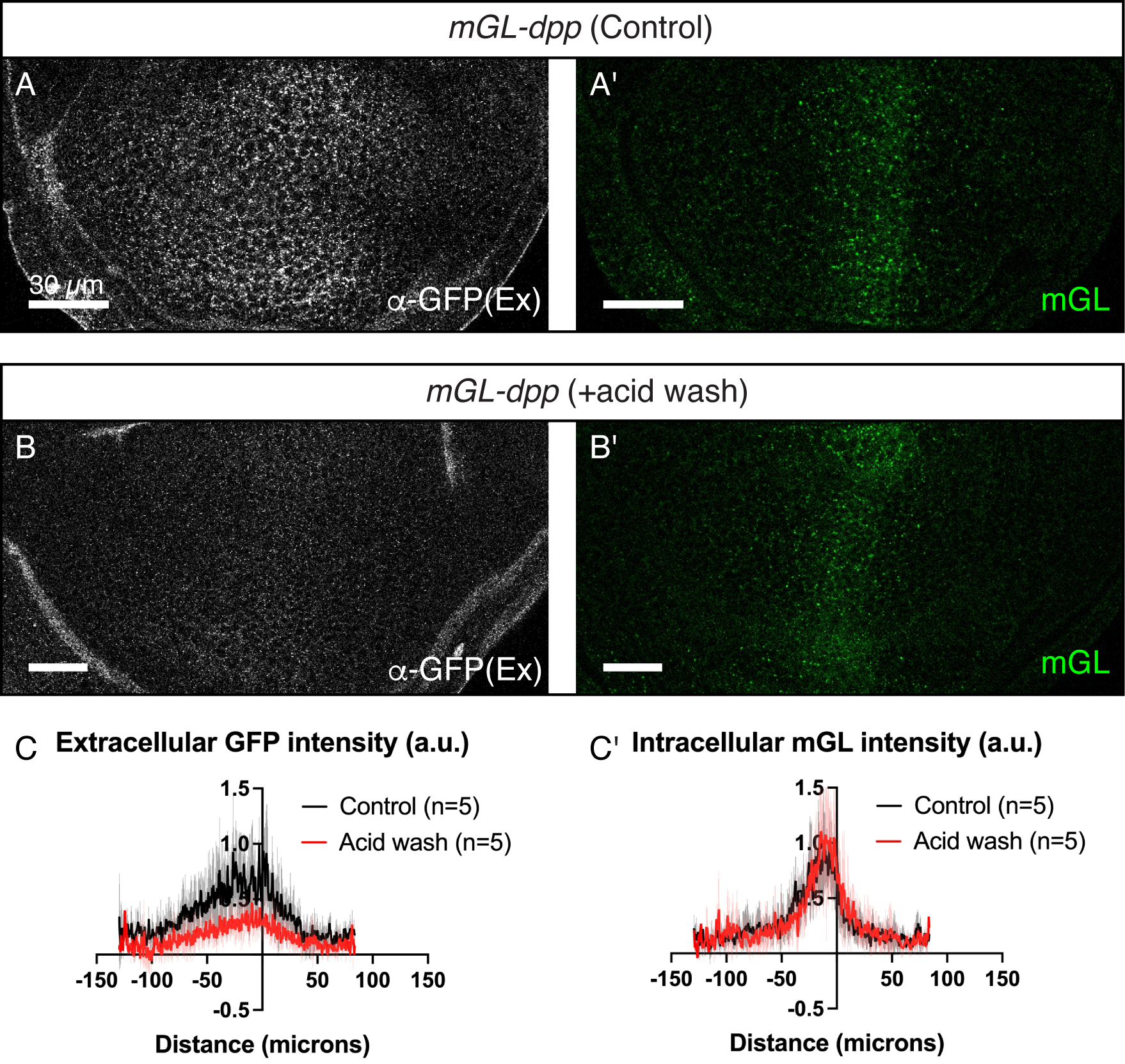

Supplement: S1 Fig — (A-A’) Extracellular α-GFP staining (A) and mGL-Dpp fluorescent signal (A’) of control wing disc without acid wash. (B-B’) Extracellular α-GFP staining (B) and mGL-Dpp fluorescent signal (B’) after the acid wash. (C-C’) Quantification of the extracellular GFP intensity of A and B (C), and the intracellular mGL fluorescent intensity in A’ and B’ (C’). (TIF) [file pgen.1011766.s001.tif]

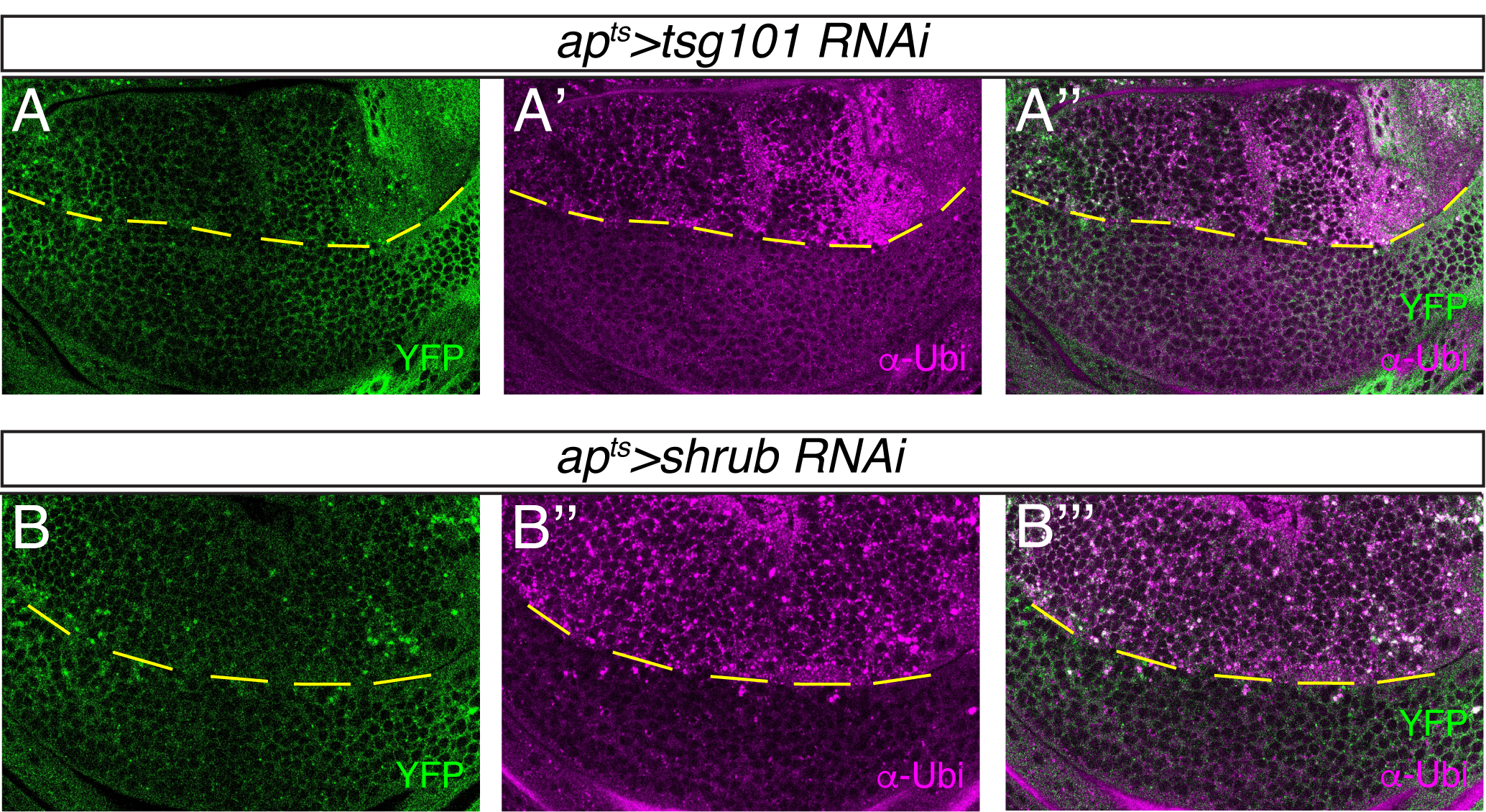

Supplement: S2 Fig — (A-A”) Tkv-YFP fluorescent signal (A), α-Ubiquitin staining (A’), and the merged image (A”) of apts > tsg101 RNAi wing disc. (B-B”) Tkv-YFP fluorescent signal (B), α-Ubiquitin (B’), and the merged image (B”) of apts>shrub RNAi wing disc. (TIF) [file pgen.1011766.s002.tif]

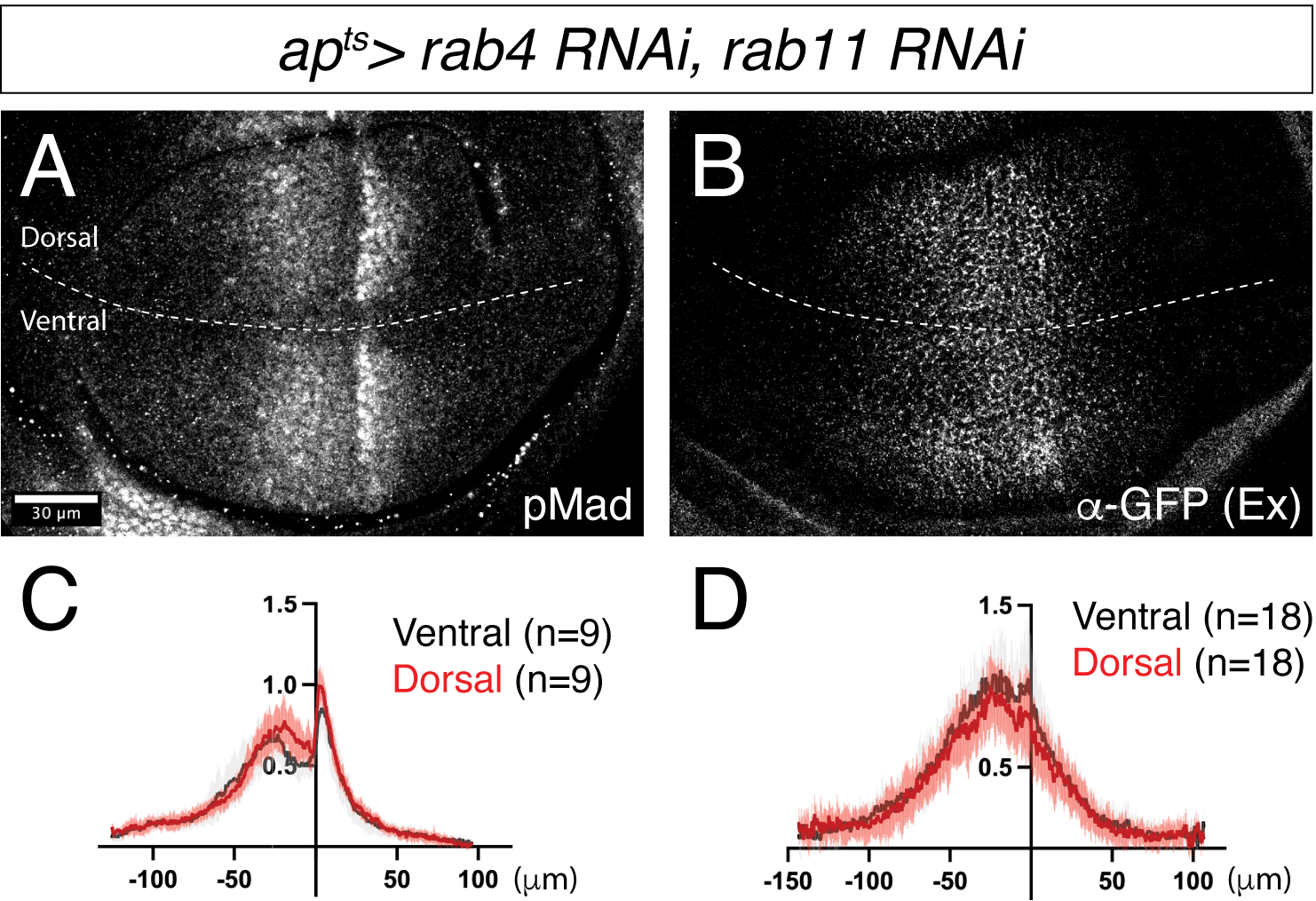

Supplement: S3 Fig — (A, B) α-pMad staining (A) and extracellular α-GFP staining (B) of apts > rab11 RNAi wing disc. (C) Average fluorescence intensity profiles of (A). Data are presented as mean + /- SD. (D) Average fluorescence intensity profiles of (B). Data are presented as mean + /- SD. Scale bar: 30μm. (TIF) [file pgen.1011766.s003.tif]
