# Supplementary material for: Shaping and interpretation of Dpp morphogen gradient by endocytic trafficking
Source: PLoS Genet. 2025 Jul 14;21(7):e1011766. doi: 10.1371/journal.pgen.1011766 (PMC12310002; doi:10.1371/journal.pgen.1011766)
Supplement: Attachment — Submitted filename: Response to reviewers comments.docx [file pgen.1011766.s007.docx]

Please find our rebuttal letter that responds to each point raised by reviewers.

The reviewers’ comments are colored in blue and our response to the comments are in black.

Reviewer's Responses to Questions

**Comments to the Authors:**

**Please note here if the review is uploaded as an attachment.**

Reviewer #1: This interesting manuscript considers a long-standing problem in the field of morphogen signalling, in particular relating to the mechanisms of DPP signalling, a key BMP morphogen, in Drosophila wing patterning. It relies on critical new tools, two lines of flies in which the endogenous DPP protein is tagged with green and red fluorescent proteins. Unlike previous endogenous fluorescently tagged Dpp lines, it is possible to visualise intracellular and extracellular DPP in wing discs from these lines and therefore assess the effects of different genetic manipulations on the endogenous protein, and this leads to some findings that contrast with experiments undertaken with overexpressed DPP.

Using these tools and some genetic tricks to allow the genetically manipulated animals to consistently survive through larval development, the authors provide evidence that Dynamin-mediated internalisation is required to activate DPP signalling. However, early endosomal Rab5 is not required either for signal activation or signal spread, though it does play a role in downregulating DPP signalling, probably through ESCRT-mediated internalisation of TKV-containing ILVs in MVBs. Rab7 knockdown experiments suggest that late endosome formation is not essential for this process.

Overall, I thought the experiments presented in the manuscript were generally of a high quality. The interpretation is inevitably limited by the fact that many of the genetic manipulations will have knock-on cell biological effects in knockdown/mutant cells, which might suppress or mask the direct effects of the manipulations. But importantly, there are differences in the effects of the different manipulations, which suggest that only some of the steps tested have a role in DPP signalling activation and signal downregulation.
I have a few suggestions that I think would help to support the conclusions made or aid clarity.

1. Figure 4: I wondered whether the authors had tried to determine the identity of basal compartments containing more DPP/TKV in Rab5 knockdown cells by using YFP-Rab markers and the red-DPP they employ. It is not clear to me where endocytosed DPP will reside if it is not trafficked to the early endosome.

Given that loss of Rab5 is expected to affect early endosome formation and following endosomal maturations, we speculate that mGL-Dpp likely reflects retention in early vesicles that fail to fuse with early endosome. However, since there is no Rabs that mark such vesicles, we could not directly test this possibility in this work. We discussed this point as follows.

“Since endocytic trafficking downstream of Rab5 is also disrupted in Rab5 mutants^36,40,41^, mGL-Dpp likely accumulates in early endocytic vesicles that fail to fuse with early endosomes.” (p.6 line 20-22).

“Loss of Rab5 resulted in increased Dpp signaling (Fig. 3) accompanied by accumulation of Dpp and Tkv likely within early endocytic vesicles that fail to fuse with early endosomes, particularly on the basal side (Fig. 4). Although the precise identity of these vesicular compartments remains unclear due to the absence of compartment-specific markers, partial degradation of Tkv was sufficient to suppress the enhanced Dpp signaling (Fig.5), suggesting that excess Tkv is required for the phenotype.” (p.8 line40-45)

2. Figure 6: More importantly, I think some more analysis is required to interpret the roles of ESCRTs versus Rab7.

There was no analysis of co-localisation of Ubi and Tkv in ESCRT knockdowns - were most of the large Tkv-containing compartments Ubi-positive or just a small fraction? Knowing this might allow us to determine whether these are mainly stalled MVB compartments.

We performed co-localization analysis of Ubi and Tkv in ESCRT knockdowns. The colocalization map (Fig. 6D’’) showed higher number of colocalized Tkv with Ubi. We also found that 80.3% of TkvYFP co-localizes with Ubi in the dorsal compartment, while 56.2% of TkvYFP co-localizes with Ubi in the ventral compartment (Fig.6D’’). Thus co-localization of TkvYFP with Ubi indeed increased. Given that majority of Tkv-YFP resides as large puncta, this result suggests that most of the large Tkv-containing vesicles are Ubi-positive.

And since DPP accumulates in large compartments in ESCRT knockdown cells, could the Rab identity of these compartments be analysed with the red-DPP marker to see whether the DPP resides in late or recycling endosomal compartments? Figure 6H’’’ is also missing, so the important data quantification is absent.

Given that ubiquitinated proteins accumulate in early endosome upon knocking down ESCRT complex, we speculate that Dpp is trapped in the early endosome to continue to activate Dpp signaling. By analysing co-localization of Rab5-eYFP with mSC-dpp upon knocking down TSG101, we found that co-localization of Rab5-eYFP and mSC-Dpp indeed increased. We added this result as Fig. 6H and described as follows (p7.line14-16). We also added the missing Fig. 6H’’’ (now as Fig. 6G).

“Consistent with the accumulation of ubiquitinated proteins in Rab5-positive compartments^44^, we observed increased co-localization of mSC-Dpp with Rab5-eYFP following TSG101 knockdown by RNAi (Fig. 6H, I).”

Finally, in the Rab7 knockdown cells, levels of TKV seem largely unaffected. But if late endosomes and lysosomes are not forming, wouldn’t TKV become trapped in accumulating MVBs? This point needs some consideration in the Discussion.

We agree with the reviewer that Tkv likely accumulates in MVBs. However, due to the lack of reliable fluorescent markers or antibodies for endogenous MVBs, we could not address this issue in this manuscript. We discuss the potential reason why the the overall distribution of Dpp and Tkv remained unchanged upon Rab7 loss as follows (p.9 line26-33).

“Since Rab7 is not required for MVB biogenesis^46^, we speculate that loss of Rab7 causes accumulation of Dpp and Tkv in ILVs without enhancing Dpp signaling (Fig. 9). Although we were unable to determine the precise subcellular localization of Dpp and Tkv due to the lack of reliable fluorescent markers or antibodies for endogenous MVBs, the overall distribution of Dpp and Tkv remained unchanged, possibly due to pH-dependent quenching of fluorophores within MVBs. Alternatively, Dpp and Tkv may still undergo degradation, as Rab7 deficiency has been reported to induce lysosome formation from recycling endosomes, thereby promoting degradation of cell surface proteins^55^.”

3. Figure 7: Figure 2 suggests there is quite strong co-localisation between DPP and Rab7. So if Rab7 is knocked down, where does that DPP now reside? Again, this could be assessed with the red-DPP and labelled Rabs. Regarding the Rab7 experiments in general, I believe it would also be useful to quote one or more previous studies that suggest Rab7 is not required for MVB biogenesis, eg. Rab7 after, eg: [https://Ipubmed.ncbi.nlm.nih.gov/19265192/](https://pubmed.ncbi.nlm.nih.gov/19265192/).

We thank the reviewer for suggesting a paper. We cited the paper and discussed the possibility about where Dpp resides upon loss of Rab7 in Discussion as we answered in the previous question (p.9 line26-33).

4. I think the authors should include a table of the effects on extracellular/intracellular, basal/lateral DPP localisation, Tkv and DPP signalling for each genetic manipulation either with Figure 9 or in a Supplementary Figure. I think this would help to contrast the different data presented.

We thank the reviewer for the suggestion. We now included a supplementary table.

5. For Figure 9, I also suggest the authors include the different genes tested at different stages of the endocytic pathway on the diagram (and potentially add in Rab4 and Rab11 recycling steps, so it is clear where they intersect with the degradative pathway).

We thank the reviewer for the suggestion. We now included the different genes in the model.

6. In the Discussion, there should at least be some reference to the fact that ESCRTs are not just involved in ILV formation, even though the interpretation that ILV blockade is how they likely function in DPP signalling downregulation seems reasonable.

We thank the reviewer for the suggestion. We added the some references and mention the other roles of ESCRT compponets as follows (p.9 line5-9).

“ESCRT components are involved in multiple cellular processes, including ILV formation^51^, nuclear envelope reformation^52,53^, and cytokinetic abscission^54^. Among these functions, we propose a model in which ESCRT machinery facilitates the sorting of ubiquitinated Tkv into ILVs, thus physically separating activated Tkv from its cytosolic effector Mad and terminating Dpp signaling (Fig. 9).”

7. In the Discussion, it is suggested that the data ‘support the idea that early endocytosis simply acts as a sink for Dpp’. I think that needs some more clarity, with ‘early endocytosis’ replaced by ‘trafficking to the early endosome’.

We thank the reviewer for the suggestion. Since we actually wanted to say that endocytosis simply acts as a sink for Dpp, we replaced ‘early endocytosis’ with ‘endocytosis’ in Discussion as follows (p.8 line21-24).

“These results argue against transcytosis or recycling as major contributors to Dpp spreading^49^ or recycling^20^ and instead support a model in which endocytosis primarily functions as a sink to remove extracellular Dpp (Fig. 9). ”

The Rab11 knockdown result suggests that endosomal and DPP trafficking is significantly disrupted in knockdown cells, but DPP signalling is unaffected. Do the authors think that DPP is accumulating in the early endosomal sink here?

Given that early endosome has been shown to be enlarged upon loss of Rab11, we speculate that Dpp accumulated in enlarged early endosome. Interestingly, loss of Rab11 has been shown to enhance subsequent late endosomal/lysosomal activity^40^ contrast with loss of ESCRT complex, which may explain why Dpp signal is rather unaffected despite the accumulation of Dpp in the enlarged early enosome. We discussed this in Discussion as follows (p.9 line33-39).

“It remains also unclear how endocytic trafficking and Dpp distribution are regulated to maintain overall Dpp levels and signaling in the absence of Rab4 and/or Rab11. Notably, Rab11 knockdown caused accumulation of Dpp likely in enlarged early endosomes on the basal side (Fig. 8M, N). However, this accumulation does not appear to enhance Dpp signaling, likely because Rab11 knockdown promotes subsequent trafficking to late endosomes and lysosomes^48^, in contrast to the trafficking blockage caused by loss of the ESCRT complex.”

Minor points

1. Figure 1: I wondered whether the authors could be certain that the GFP antibody employed does not affect GFP fluorescence by referring to previous studies using this antibody?

We found a previous study using this antibody to show that the GFP antibody does not affect GFP fluorescece (<https://www.nature.com/articles/s41594-021-00560-2/figures/11>). We cited this paper in the result section as follows (p.4 line43-46).

“To determine whether the mGL-Dpp fluorescent signal originates from extracellular or intracellular Dpp, we performed extracellular staining using an α-GFP antibody, which recognizes mGL (like GFP, as both are derived from *Aequorea victoria*) and does not interfere with GFP fluorescence^34^.”

2. Statistics – all the statistics use a t-test, which assumes parametrically distributed data points. Has any test been undertaken to confirm that this is the case (this should be mentioned), therefore showing that a non-parametric test is not required?

We performed the normality tests for all the statistics and provided the results as supplements. For some tests, we could not confirm parametric distribution and thus used non-parametric tests for such cases.

3. Although generally well-written, there are a number of minor typos throughout the text, eg.
Page 3, near bottom of last paragraph: ‘the duration of Dpp signalling affects interpretation of the extracellular …’
Page 5, bottom title – ‘Rab5-mediated’
Figure 1 legend: ‘anti-GFP staining’ or ‘α-GFP’, ‘50 µm’

We thank the reviewer for pointing out the typos. We corrected them.

Reviewer #2: This manuscript by Rasouliha and colleagues examines the cellular mechanisms controlling Dpp signaling gradients in the Drosophila fly wing using molecular genetic and cellular approaches. A major strength of the study is that the cell biological experiments are done in an in vivo (albeit dissected) context, which adds considerable value (and difficulty) to the work. Some of this was facilitated by the generation of endogenously tagged alleles of Dpp, which may be useful to the field. Another strength and point of interest is the focus on cell signaling and morphogen gradients, including data that contradicts some of what has been previously reported for mechanisms controlling Dpp signaling and gradients. In the end, I found the central model, that most Dpp signaling (pMad activation) occurs intracellularly following Dpp internalization but before quenching in MVBs, to be convincing. Perhaps less clear was how intracellular trafficking pathways impact extracellular Dpp accumulation or why ESCRT seems to affect Dpp signaling differently than rab5, although these are more minor points.

Major issue:

In general, this paper needs substantial grammatical/English editing throughout to improve clarity, readability, and correct errors including some typos. In the end I was able to work through most of the sections and gain a sufficient understanding of what the authors meant to convey, which allowed me to review the science. Still, there were numerous sentences that I ultimately remained confused about. As such, the writing needs considerable work, preferably with the aid of a professional copy editor or equivalent. Doing this prior to submitting is preferrable. I also thought that several sections would benefit from more substantive background information and explanations, which many readers would require regardless of grammar. To me the writing was overly terse.

We are sorry for the grammatical/English errors and typos. We used OpenAI’s ChatGPT to assist with language editing and proofreading of this manuscript. In addition, we followed the reviewer’s suggestions to add background information and explanations for non-experts. We hope that the manuscript has been improved.

Below are specific points relevant to each section of the manuscript for the authors to consider.

1) Figure 1/S1 and associated text.

i) Non-fly readers may benefit from an example of WT to accompany D/E.

We thank the reviewer for the suggestion. We added an control wing image as Fig. 1D.

ii) Here and throughout, certain terms and descriptions were somewhat confusing. For example, “Dpp signal” means pMad (phosphorylation) whereas “mGL-Dpp signal” means the fluorescently tagged Dpp protein.

We are sorry for the confusion. We changed “mGL-Dpp signal” to “mGL-Dpp fluorescent signal” or “mGL-Dpp distribution” depending on the contexts.

iii) State that anti-GFP antibodies recognize mGL, from which it is derived.

We mentioned that anti-GFP antibodies recognize mGL derived from Aequorea victoria like GFP as follows (p.4 line43-46).

“To determine whether the mGL-Dpp fluorescent signal originates from extracellular or intracellular Dpp, we performed extracellular staining using an α-GFP antibody, which recognizes mGL (like GFP, as both are derived from *Aequorea victoria*) and does not interfere with GFP fluorescence^34^.”

iv) I was somewhat confused by the anti-GFP and mGL co-localization study. Lack of co-loc or apparent correlation in these expression patterns seems surprising since they are looking at the same molecule, even if one shows both intra and extracellular expression and the other extracellular only. In addition, the gradients of extracellular Dpp seemed very shallow (visually) relative to pMad and intracellular Dpp. Some explanations here seem warranted.

We are sorry for the confusion. As the total mGL-Dpp fluorescent signal was not affected by the acid wash, which efficiently removes extracellular proteins (Fig. S1), the majority of total mGL-Dpp fluorescent signal reflects intracellular Dpp. This can explain why total mGL fluorescent signal rarely colocalizes with extracellular α-GFP signal. Furthermore, since extracellular staining amplifies weak or even subdetection level of the mGL-Dpp fluorescence to reveal extracellular Dpp gradient, extracellular α-GFP signal does not always colocalize with total mGL fluorescent signal. We added these explanation in Result section as follows (p5. Line1-7).

“Interestingly, the extracellular α-GFP distribution and total mGL-Dpp fluorescence rarely colocalized (Fig. 1L’’). Since the total mGL-Dpp fluorescent signal was not sensitive to acid wash, which efficiently removes extracellular proteins^20^ (Fig. S1), the majority of the mGL-Dpp fluorescent signal likely originates from intracellular Dpp. Furthermore, the extracellular staining amplifies weak or even sub-detection levels of the total mGL fluorescence to reveal extracellular Dpp distribution. Together, there factors may explain the limited overlap between the mGL fluorescence and the extracellular α-GFP signal.”

In addition, we discussed why the extracellular mGL-Dpp gradient appears shallower than intracellular Dpp gradient and Dpp signaling gradient in Result section as follows (p.4 line49-53).

“The elevated total signal in the center likely reflects intracellular Dpp within the secretory pathway of Dpp-producing cells. Interestingly, the Dpp signaling gradient appears steeper than the extracellular mGL-Dpp gradient, suggesting a threshold-dependent response to extracellular Dpp required for signaling activation.”

v) S1A and 1K’ look rather different although they are both anti-GFP of mGL-Dpp. The somewhat graded expression in S1A, with higher levels in the center, also correlates more mGL, which is what one might expect. I wasn’t sure what to make of this.

Although both S1A and 1K’ (new 1L’) are extracellular GFP staining of mGL-Dpp, Fig.1K’ (new 1L’) is a single confocal section to focus on co-localization of anti-GFP signal with mGL fluorescent signal, and Fig. S1A is an stack of averaged three sequential confocal sections along the apical-basal axis to visualize the overall graded distribution of extracellular mGL-Dpp. Thus, S1A shows smoother extracellular Dpp gradient with higher signal in the center of the wing disc than 1K’ (new 1L’).

2) Figure 2 and associated text.
i) Might want to clarify that the Mander’s overlap adds up to ~150% probably because of some overlap in the trafficking markers or just coincidental co-loc.

Each image was quantified separately and they are not supposed to all add up to 100%. As the reviewer mentioned, this may be due to an overlap of the trafficking markers themselves. For example, in some instances, Dpp-mSC can colocalize with both Rab5 and Rab7, as a maturing endosome has both Rab5 and Rab7 and Rab5 is being replaced by Rab7. Also, Rab7 can be present on both maturing endosome as well as the late endosome.

ii) Not here, but later it becomes somewhat confusing that recycling doesn’t seem to play a role in Dpp expression despite the observed co-loc to rab4 and rab11 compartments.

We thank the reviewer for the comment. We do not currently know how endocytic trafficking is affected and in which compartments Dpp is redistributed upon loss of Rab4 or/and Rab11 but we speculate that loss of colocalization of Dpp with recycling endosomes was compensated by the changes in endocytic trafficking and Dpp redistribution such that Dpp signal was not affected. We discussed this point in Discussion as follows (p.9 line33-39).

“It remains also unclear how endocytic trafficking and Dpp distribution are regulated to maintain overall Dpp levels and signaling in the absence of Rab4 and/or Rab11. Notably, Rab11 knockdown caused accumulation of Dpp likely in enlarged early endosomes on the basal side (Fig. 8M, N). However, this accumulation does not appear to enhance Dpp signaling, likely because Rab11 knockdown promotes subsequent trafficking to late endosomes and lysosomes^48^, in contrast to the trafficking blockage caused by loss of the ESCRT complex.”

3) Figure 3 and associated text.

i) It might be useful to provide more complete genotypes in the figure legends that would include gal80, which was specifically mentioned in the text.

We thank the reviewer for the suggestion. Since *ap^ts^* is repeatedly used in other figures and figure legends, we decided to introduce *ap^ts^* as *ap*-Gal4 with the temperature-sensitive Gal80 (Gal80ts) and to use *ap^ts^* in this ms as follows (p.5 line28-34). We also explained Gal80ts system for non-fly experts.

“To test this, we used *ap*-Gal4 combined with the temperature-sensitive Gal80 (Gal80ts), hereafter referred to as *ap^ts^* for simplicity, to temporally express UAS-Rab5 RNAi in the dorsal compartment of the wing discs (*ap^ts^>Rab5RNAi*) (Fig. 3D). To minimize the pleiotropic effects associated with Rab5 knockdown, Gal80ts enables precise temporal control of gene expression through temperature shifts: at 18 °C, Gal80ts binds and inhibits GAL4 activity, while at 29 °C, Gal80ts becomes inactive, allowing Gal4 to drive transcription from UAS sites.”

ii) Please indicate the dorsal compartment for at least one of the panels. This would be useful in several other figures as well. Many readers won’t know what they are looking at or where the ap driver is active.

We indicated the dorsal compartment in some of the panels in Fig.3 and other Figs. We also added schematic image of the wing disc in Fig. 1 for non-fly experts.

4) Figure 4 and associated text.

i) I was initially confused by the wording introducing the experiment shown in 4A. Again, given that this is a general audience and that there are no word limits, it would be helpful to explain some of the experiments more fully.

We are sorry for the confusion. To explain the experiment more fully, we rewrote the part as follows (p5. Line46-52).

“To investigate how loss of Rab5 leads to increased Dpp signaling, we first asked whether this increase is dependent on Dpp itself. Because *dpp* mutants lack wing tissue, we took advantage of the fact that Dpp promotes wing disc growth primarily by repressing the growth inhibitor Brk, and that *dpp, brk* double mutants can grow in the absence of Dpp signal^37-39^. When Rab5 was knocked down in a *dpp, brk* mutant background, Dpp signaling was not upregulated, indicating that the observed increase in Dpp signaling was indeed Dpp-dependent (Fig. 4A).”

ii) I wasn’t given sufficient information or background to understand the meaning of the results in 4I-P or to know why the specific conclusion was made.

We are sorry for the confusion. Given that loss of Dpp signaling in *shi^ts^* mutants suggests that internalized Dpp is required for signaling activation (Fig. 3B, 4F-H), we asked whether intracellular Dpp distribution is altered upon loss of Rab5. While Dpp and Tkv distribution reduced from lateral side likely because Rab5 impaires endocytosis, we found that Dpp and Tkv accumulated in basal side. Since endocytic trafficking downstream of Rab5 is disrupted in Rab5 mutants^36,40,41^, Dpp and Tkv are likely trapped in early endocytic vesicles that fail to fuse with early endosome. We reformulated the paragraph as follows (p6. line15-24).

“Given that internalized Dpp is required for signaling activation (Fig. 3B, 4F-H), we next asked whether intracellular Dpp distribution is altered upon loss of Rab5. As expected from impaired endocytosis, Rab5 knockdown appeared to reduce the number of intracellular mGL-Dpp puncta laterally, although this reduction was not statistically significant (Fig. 4I, M). In contrast, Rab5 loss led to a significant increase in mGL-Dpp puncta at the basal side of the wing disc (Fig. 4J, N). Since endocytic trafficking downstream of Rab5 is also disrupted in Rab5 mutants^36,40,41^, mGL-Dpp likely accumulates in early endocytic vesicles that fail to fuse with early endosomes. Similarly, Tkv-YFP showed reduced lateral distribution particularly in the posterior compartment and accumulated basally in peripheral regions upon Rab5 knockdown (Fig. 4J, K, N, O). ”

iii) Looking at the images in K and L I had a hard time seeing discrete puncta and thus wondered about the quantification using # of puncta in O and P.

We thank the reviewer for the comment. We replaced the quantification using # of puncta with the average fluorescence intensity profile.

5) Figure 5 and associated text.

i) I was a bit puzzled as to why the dorsal expression of pMad was relatively unaffected in the WT background versus the rab5 rnai. To my eye, the level of TKv-eGFP in the dorsal half of the 5C disc was clearly lower than the ventral side. But that was less the case for 5D. If knockdown was less in 5D for some reason, that could explain this discrepancy.

We are sorry for the confusion. As the reviewer mentioned in the next question, we agree that these results simply indicate that increased Dpp pathway activation following rab5 inhibition is dependent on the Dpp receptor, Tkv. Especially, since partial degradation of one copy of Tkv-HA-eGFP by degradHA does not siginificantly affect pMad gradient in wild-type background but rescue the increase of Dpp signal caused by loss of Rab5, our interpretation is that increased Dpp pathway activation following rab5 inhibition is dependent on excess Tkv resulting from Rab5 inhibition.

ii) The final concluding sentence, “These results suggest that Dpp signaling is not terminated in the absence of Rab5 due to impaired downregulation of activated Tkv.”, was confusing to me. This is one example of my major point above. Dpp signaling is shown to be increased in the absence of rab5, so the use of “terminated” was puzzling in this context. To me, these results show that increased Dpp pathway activation following rab5 inhibition is dependent on the Dpp receptor, Tkv. Is there something that I’m missing? Regardless, the sentence did not clearly summarize the result or its interpretation.

We are sorry for the confusion. We agree with the reviewer that these results show that increased Dpp pathway activation following rab5 inhibition is dependent on the Dpp receptor, Tkv. We removed the final concluding sentence and changed the title of the paragraph, and rewrote the paragraph accordingly. Especially, as we also mentioned for the above question, since partial degradation of one copy of Tkv-HA-eGFP by degradHA does not siginificantly affect pMad gradient in wild-type background but rescue only the increase of Dpp signal caused by loss of Rab5, our interpretation is that increased Dpp pathway activation following rab5 inhibition is dependent on excess Tkv resulting from rab5 inhibition.

6) Figure 6/S2 and associated text.

i) “We then asked in which endocytic trafficking Dpp signal is terminated?” Another example of lack of clarity and grammatical issues. A corrected sentence might read, “We next asked in which endocytic trafficking compartment Dpp signaling is terminated.” Please have this manuscript edited.

We thank the reviewer for pointing out this. We corrected the sentence as follows(p.6 line49-50).

“But in which endocytic trafficking compartment is Dpp signal terminated downstream of Rab5?”

ii) Although the results were generally quite clear from the images, I might have expected somewhat more quantification of the data (e.g., the difference between H’ and H”).

We added the missing Fig. 6H’’’ ( Fig. 6G in the revised ms) to show that the size of mGL-Dpp puncta increased in vps4 RNAi wing disc. Based on the reviewer1’s request, we also performed experiments to test whether Dpp is accumulated in the early endosome to continue to activate Dpp signaling in ESCRT mutants. By analysing co-localization of Rab5-eYFP with mSC-dpp upon knocking down TSG101, we found that co-localization of Rab5-eYFP and mSC-Dpp indeed increased. We added this result as Fig. 6H and quantification as Fig. 6I.

iii) Is the idea that Dpp/receptors are ubiquitylated or that inhibiting cargo sorting into MVBs leads to the general accumulation of ubiquitylated proteins in cells?

The idea is that inhibiting cargo sorting into MVBs leads to the general accumulation of ubiquitylated proteins including Tkv.

iv) It appears that the effect of ESCRT rnai on pMad greater than that observed for rab5 rnai? If so, why would that be?

Since loss of Rab5 affects endocytosis (Fig.4C) whereas loss of ESCRT complex does not (new Fig. 6I, J), the different effects on pMad is probably because internalized Dpp (contributing to signaling) is reduced in Rab5 RNAi discs compared to ESCRT RNAi discs. We discussed this in Discussion (p.9 line10-14).

“Notably, we observed that loss of the ESCRT complex results in a greater increase in Dpp signaling than loss of Rab5 (Fig. 3D, G, H, Fig. 6A–C). Since Rab5 loss impairs Dpp internalization (Fig. 4C), whereas loss of the ESCRT complex does not disrupt endocytosis (Fig. 6J, K), the differential effects on Dpp signaling likely reflect the reduced internalized Dpp available for signaling in Rab5 RNAi discs compared to ESCRT RNAi discs.”

7) Figure 7 and associated text.

i) What is the explanation for the lack of any difference in mGL-Dpp when rab7 is inhibited? Has endosomal Dpp been degraded despite rab7 knockdown? Has the pH dropped sufficiently within endosomes/MVBs to quench the mGL signal?

We thank the reviewer for the question. We discussed possible explanation why mGL-Dpp distribution was not affected upon loss of Rab7 in Discussion as follows (p.9 line26-33).

“Since Rab7 is not required for MVB biogenesis^46^, we speculate that loss of Rab7 causes accumulation of Dpp and Tkv in ILVs without enhancing Dpp signaling (Fig. 9). Although we were unable to determine the precise subcellular localization of Dpp and Tkv due to the lack of reliable fluorescent markers or antibodies for endogenous MVBs, the overall distribution of Dpp and Tkv remained unchanged, possibly due to pH-dependent quenching of fluorophores within MVBs. Alternatively, Dpp and Tkv may still undergo degradation, as Rab7 deficiency has been reported to induce lysosome formation from recycling endosomes, thereby promoting degradation of cell surface proteins^55^.”

8) Figure 8 and associated text.

i) Is it possible that Dpp can be recycled by either slow (rab11) or fast (rab4) recycling pathways and that both would have to be compromised before a strong change in the Dpp gradient was observed? This seems like a possibility given that Dpp localized to both compartments. Is it feasible to knock down both pathways simultaneously to see how this impacts Dpp?

To test whether Rab4 and Rab11 act redundantly, we simultaneously knocked down Rab4 and Rab11 by RNAi and found that Dpp signaling and extracellular mGL-Dpp distribution were not significantly affected (Fig. S3).

9) Figure 9 and Discussion.

i) Consider including additional diagrams in Fig 9 to visualize what is happening in some of the mutant backgrounds with respect to signaling and Dpp localization.

We thank the reviewer for the suggestion. We included Dpp signaling and localization in different mutants in Fig.9.

ii) (Repeated from above) Is the effect of ESCRT rnai on pMad greater than rab5 rnai? If so, why would that be with respect to the provided model.

We answered this question above.

As for all my reviews this is signed by David Fay

Thank you very much for the constructive comments!
