# Supplementary material for: Shaping and interpretation of Dpp morphogen gradient by endocytic trafficking
Source: PLoS Genet. 2025 Jul 14;21(7):e1011766. doi: 10.1371/journal.pgen.1011766 (PMC12310002; doi:10.1371/journal.pgen.1011766)
Supplement: Attachment — Submitted filename: Response to Reviewers.docx [file pgen.1011766.s008.docx]

Response to Reviewers

Please find our rebuttal letter that responds to each point raised by reviewers.

The reviewers’ comments are colored in blue and our response to the comments are in black.

Reviewer #1: The authors have addressed my comments in their revised version of the manuscript. Thank you.

Thank you very much again for the constructive comments on our manuscript.

I only have two minor comments:
1. I think the Supplementary Table is helpful, but I couldn’t see a reference to it in the text – perhaps at the start of the Discussion?

We added the following sentence at the start of the Discussion.

“An overview of the results is summarized in the Supplementary Table (Supplementary Table).”

2. page 9, line 26 needs rewording, ie: ‘we speculate that loss of Rab7 causes increased association of Dpp and Tkv with ILVs….’, since Dpp is likely to be outside ILVs not in them.

Thanks. We corrected the sentence as follows.

“Since Rab7 is not required for MVB biogenesis^46^, we speculate that loss of Rab7 causes accumulation of Dpp and Tkv outside ILVs without enhancing Dpp signaling (Fig. 9).”

Reviewer #2: The authors have done a very good job of addressing prior concerns and the manuscript is substantially improved. It's a very nice story. David Fay

Thank you very much again for your constructive comments!
